# GP或TP化疗联合热疗治疗非小细胞肺癌的系统评价

**DOI:** 10.3779/j.issn.1009-3419.2012.08.02

**Published:** 2012-08-20

**Authors:** 登海 米, 征 李, 克虎 杨, 农 曹, 金徽 田, 彬 马

**Affiliations:** 1 730000 兰州，兰州大学循证医学中心 Evidence Based Medicine Center of Lanzhou University, Lanzhou 730000, China; 2 730000 兰州，兰州大学第一临床医学院普外科 Department of General Surgery, the First Clinical Medicine College of Lanzhou University, Lanzhou 730000, China; 3 730000 兰州，甘肃省第二人民医院肿瘤科 Department of Oncology, the Second People's Hospital of Gansu Province, Lanzhou 730000, China

**Keywords:** 肺肿瘤, 热疗, 化疗, 顺铂, 吉西他滨, 紫杉醇, *Meta*分析, 系统评价, 随机对照试验, Lung neoplasms, Hyperthermia, Chemotherapy, Cisplatin, Gemcitabine, Paclitaxel, *Meta*-analysis, Systematic review, Randomized controlled trial

## Abstract

**背景与目的:**

中晚期非小细胞肺癌（non-small cell lung cancer, NSCLC）的治疗效果较差，生存期也较短，临床研究显示化疗与热疗的联合疗法效果较好。本研究通过系统评价吉西他滨联合铂类（Gemcitabine and Cisplatin, GP）或紫杉醇联合铂类（Paclitaxel and Cisplatin, TP）方案化疗联合热疗治疗NSCLC的疗效及安全性，为临床实践与研究提供参考。

**方法:**

计算机检索Cochrane Library、PubMed、EMBASE和中国生物医学文献数据库、中国期刊全文数据库、中文科技期刊数据库、数字化期刊全文数据库，同时辅以其它检索。收集所有比较GP或TP化疗联合热疗与单纯GP或TP化疗的随机对照试验（randomized controlled trial, RCT）。选择适当的质量评价标准对纳入文献进行质量评价，使用RevMan 5.1软件进行*meta*分析。

**结果:**

共纳入15篇RCT，共952例NSCLC。*Meta*分析结果显示，化疗联合热疗组的生活质量改善率（OR=3.84, 95%CI: 2.61-5.64）、1年生存率（HR=1.94, 95%CI: 1.21-3.12）和2年生存率（HR=2.05, 95%CI: 1.18-3.58）均好于单纯化疗组，差异均有统计学意义（*P* < 0.05）；热化组与单化组的骨髓抑制、胃肠反应、肝肾损害以及腹泻发生率的差异均无统计学意义（*P* > 0.05）。

**结论:**

相较于单纯化疗，GP或TP方案化疗联合热疗能提高中晚期NSCLC患者生存率和近期疗效，改善症状，提高患者的生活质量，且并不增加毒副反应，但本文结论尚需大样本高质量RCT进一步验证。

## Abstract (en)

**Background and objective:**

Advanced non-small cell lung cancer (NSCLC) is characterized by poor treatment efficacy and short survival time. Clinical trials have shown that the combination of chemotherapy with thermotherapy exhibits strong efficacy. We performed this *meta*-analysis to evaluate the clinical efficacy and safety of gemcitabine plus cisplatin (GP) and paclitaxel plus cisplatin (TP) combined with thermotherapy in the treatment of NSCLC, as well as to provide reference for clinical practice and future research.

**Methods:**

We searched international (Cochrane Library, PubMed, and EMBASE) and Chinese (CBM, CNKI, VIP and Wanfang) databases for relevant articles and imported other retrievable sources, such as tracing-related references. We also corresponded with other authors to obtain certain inaccessible information. Data from all relevant randomized controlled trials (RCT) were collected to compare GP or TP thermochemotherapy with GP or TP chemotherapy alone. The quality of the included studies was assessed by adequate outcome-based standards and clinical circumstances. The *meta*-analysis was conducted using RevMan 5.1.

**Results:**

Fifteen RCTs involving 952 patients were included in this *meta*-analysis. The results showed that the thermochemotherapy group had higher rates of improvement in quality of life (OR=3.84, 95%CI: 2.61-5.64), survival at 1 year (HR=1.94, 95%CI: 1.21-3.12), and survival at 2 years (HR=2.05, 95%CI: 1.18-3.58) compared with the chemotherapy group, with the differences between them being significant. However, these groups did not differ in other indicators of treatment effectiveness, such as myelosuppression, alimentary canal reactions, hepatic lesions, and diarrhea.

**Conclusion:**

Compared with chemotherapy alone, thermochemotherapy can improve survival rates and curative effects, ameliorate symptoms, and enhance the quality of life of patients with advanced NSCLC, and it has an acceptable safety profile. The results of this *meta*-analysis warrant further investigation with a larger sample size and using a high-quality RCT design.

肺癌是严重危害人类健康的疾病，据2008年全球最新统计数据^[1]^显示，肺癌的发病率和死亡率在男性肿瘤患者中均居首位，女性患者中肺癌发病率在肿瘤中排第四位，病死率在肿瘤中排第二位；肺癌在所有疾病中所占比例为13%（1, 600万），死亡人数在所有疾病中所占比例为18%（1, 400万）。在中国肺癌是患病率和死亡率最高的肿瘤病种，而其中非小细胞肺癌（non-small cell lung cancer, NSCLC）占全部肺癌病例的80%左右。

临床Ⅰ期-Ⅱ期NSCLC患者手术治疗的5年生存率约为40%，70%以上患者在初诊时已错过手术时机，所以非手术的其它治疗手段非常重要。目前多种治疗模式均在探讨中，化疗是治疗中晚期肺癌的重要方法之一，联合化疗已成为延长生存期、改善生活质量的重要手段，但其疗效仍不甚理想且毒副作用较大。如何提高化疗疗效并降低毒副反应是亟待解决的临床问题。

近年来肿瘤热疗学发展迅速，柳叶刀的1篇综述^[2]^充分肯定了热疗在肿瘤治疗中的价值并详细介绍了热疗的临床应用。肿瘤热疗是肿瘤综合治疗的一种重要手段，大量研究表明化疗联合热疗有明显的互补协同增效的作用，化疗与热疗联合疗法治疗NSCLC可以延长患者生存时间并提高患者生活质量。理论与临床实践都表明化疗联合热疗有良好的应用前景，但目前对该治疗模式尚缺乏相关系统评价的指导。本研究旨在评价化疗联合热疗对比单纯化疗治疗NSCLC的疗效和安全性，以期为临床实践与研究提供参考。

## 1 资料与方法

### 1.1 文献纳入标准

#### 1.1.1 研究类型

随机对照试验，无论是否隐藏或采用盲法，语种不限。

#### 1.1.2 研究对象

确诊为NSCLC且不宜手术的中晚期患者。

#### 1.1.3 干预措施

GP方案（吉西他滨+顺铂，GEM+DDP）或TP方案（紫杉醇+顺铂，TAX+DDP）全身化疗联合热疗与单纯全身化疗对比，每个随机对照试验（randomized controlled trial, RCT）中试验组与对照组之间所用化疗方案完全相同。热疗是指利用物理方法（如热疗仪）来实施加热，使肿瘤组织达到特定的温度范围（目标温度一般为41 ℃-43 ℃）并维持一定时间，利用肿瘤热效应并常配合放化疗协同来杀灭癌细胞，从而抑制和预防肿瘤复发、转移的治疗方法。

#### 1.1.4 测量指标

① 远期疗效：生存率；②近期疗效：完全缓解、部分缓解、总有效率、症状改善率、生活质量改善率；③毒副反应：并发症及不良反应发生率。

### 1.2 检索策略

#### 1.2.1 检索词

中文检索词为（非小细胞肺癌）AND（化疗）AND（热疗）AND（随机对照试验），英文检索词为（non-small cell lung cancer）AND（chemotherapy）AND（thermotherapy）AND（randomized controlled trial）。

#### 1.2.2 检索策略

检索分目标疾病和干预措施两个组面，每个组面的检索均采用主题检索与非主题检索相结合的方式，所有检索策略通过多次预检索后确定，检索词根据具体数据库进行调整，随机对照试验的检索参照Cochrane系统评价手册推荐的检索策略。为提高查全率同时从相关文献（尤其是相关综述）的参考文献中进行追溯查找。

#### 1.2.3 计算机检索

计算机检索Cochrane Library、PubMed、EMBASE、中国生物医学文献数据库（CBM）、中国期刊全文数据库（CNKI）、中文科技期刊数据库（VIP）和数字化期刊全文数据库（WanFang），为尽量避免发表偏倚，同时检索会议论文及学位论文等灰色文献数据库，收集所有化疗联合热疗治疗NSCLC的随机对照试验，检索时限为各数据库建库起至2012年5月21日，检索语种不限。

#### 1.2.4 其它检索

手检化疗联合热疗治疗NSCLC的相关文献以及相关文献的参考文献，并用Google Scholar、Medical Martix等搜索引擎在互联网上查找相关的文献。同时与本领域的专家、相关文献的通讯作者等联系以获取以上检索未发现的相关信息。

### 1.3 文献筛选和资料提取

文献筛选和资料提取由2位研究员独立操作并交叉核对。根据预先制定的纳入排除标准筛选文献，阅读所获文献题目和摘要，排除明显不符合纳入标准的文献后，对可能符合纳入标准的文献阅读全文，进一步确定是否符合纳入标准。交叉核对纳入文献的结果，对是否纳入而有分歧的文献，通过讨论并由第3位研究员决定是否纳入。

对符合标准纳入的文献进行资料提取，填写资料提取表。提取资料包括：题目、作者、发表日期、文献来源等一般资料；入选标准和样本量，抽样和分组的方法，研究对象的基本资料，研究的条件，干预的内容，测量指标，随访持续时间，病例失访率和失访的原因，统计学方法，生存率，完全缓解，部分缓解，总有效率，症状改善率，生活质量改善率，相关不良反应及并发症的发生率等结局指标数据。资料提取过程如遇分歧，通过讨论解决，对有分歧而难以确定的问题由第3位研究员进行裁定。缺乏的资料通过电话或邮件等方式与作者联系进行补充。

### 1.4 文献质量评价

结合本研究特点参考适当的标准进行质量评价：①随机分配方法，即随机序列的产生方法；②分配隐藏的实施情况；③基于结局指标的基线情况，是否具有良好的可比性；④是否采用了盲法；⑤纳入研究结果数据的完整性，对失访情况的报道。本文纳入研究干预措施涉及热疗仪，对受试者和实施干预者的盲法难以实施，但是仍可以对结果测量者和统计分析人员施盲，以求尽可能避免偏倚。

### 1.5 统计方法

采用Cochrane协作网提供的RevMan 5.1版软件进行*meta*分析。本文对GP与TP方案独立进行*meta*分析，并对其中同质性较好的指标进行合并。各纳入研究结果间的异质性采用χ^2^检验。如各研究间有统计学同质性（*P* > 0.1, *I^2^* < 50%），采用固定效应模型进行分析；如各研究间存在统计学异质性（*P* < 0.1, *I^2^* > 50%），分析其异质性来源，根据可能导致异质性的因素进行亚组分析，当亚组内各研究之间及亚组间有足够相似性时（亚组*P* > 0.1, *I^2^* < 50%）则用固定效应模型做*meta*分析，若纳入研究各亚组之间存在统计学异质性而无临床异质性或差异无统计学意义时，采用随机效应模型进行分析。如两组间异质性过大，则采用描述性分析。必要时采用敏感性分析检验结果的稳定性。

## 2 结果

### 2.1 文献检索结果

见图 1。初检出相关文献248篇，通过Endnote文献管理软件去重68篇；通过阅读题目与摘要排除115篇，初步纳入文献65篇并阅读全文；精细阅读全文后排除50篇，最终纳入15项RCT，其中SCI英文文献1篇，中文文献14篇，共952例患者。

**1:**
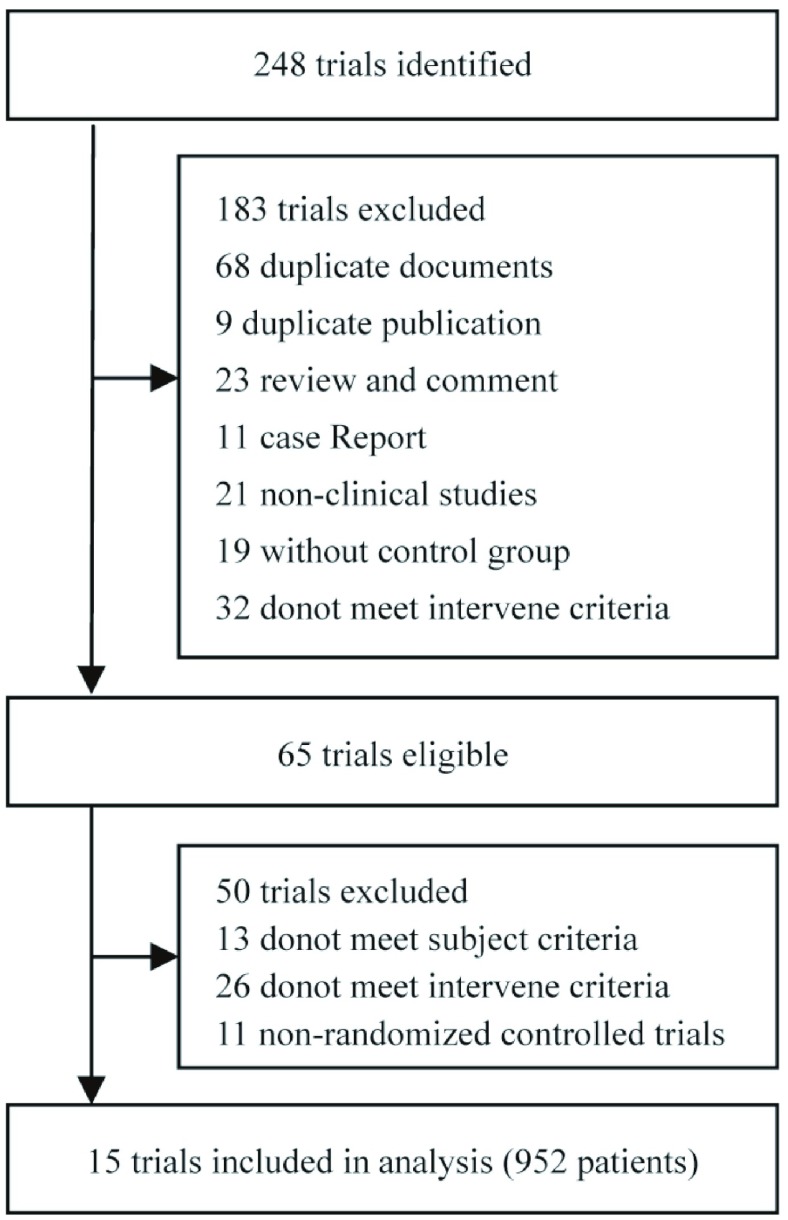
文献筛选流程及结果 Selection of trials

### 2.2 纳入研究的一般情况和质量评价

最终纳入15项RCT^[3-17]^，各研究的试验组和对照组中患者年龄、性别、临床分期等差异无统计学意义，组间基线一致，符合纳入标准，具有良好的可比性。纳入肺癌患者均为中晚期，大部分研究纳入病例卡氏评分均在60分以上，预计生存期在3个月以上。仅有2项研究报告了随机序列的产生方法，所有研究均未报告随机分配隐藏的实施情况，有2项研究的失访情况不清楚。纳入研究的特征和质量评价见表 1。

**1:** 纳入研究的一般情况和质量评价 Assessment characteristics and methodologic quality of included studies

Study (year)	Cases	Stage	KPS	Regimen	Quality assessment of methodology				
					Randomization	Allocated Concealment	Baseline control	Blinding	Loss of Follow-up
Shen H 2011^[3]^	80	Advanced	Unclear	GP	Unclear	Unclear	Adequate	Unclear	No
Zhang GZ 2011^[4]^	76	Advanced	≥60	GP	Unclear	Unclear	Adequate	Unclear	No
Chen P 2010^[5]^	52	Advanced	≥60	TP	Unclear	Unclear	Adequate	Unclear	No
Liu AH 2010^[6]^	60	Advanced	≥60	TP	Unclear	Unclear	Adequate	Unclear	No
Wang M 2010^[7]^	76	Advanced	≥50	TP	Unclear	Unclear	Adequate	Unclear	No
Yang MX 2010^[8]^	60	Advanced	≥60	TP	Unclear	Unclear	Adequate	Unclear	No
Zhang B 2010^[9]^	100	Advanced	≥60	TP	Random table	Unclear	Adequate	Unclear	Unclear
Kan SF 2009^[10]^	80	Advanced	≥60	GP	Unclear	Unclear	Adequate	Unclear	Unclear
Cao YL 2008^[11]^	64	Advanced	≥60	GP	Unclear	Unclear	Adequate	Unclear	No
Zhang L 2008^[12]^	40	Advanced	≥60	TP	Random table	Unclear	Adequate	Unclear	No
Zhou M 2007^[13]^	51	Advanced	≥60	TP	Unclear	Unclear	Adequate	Unclear	No
Chen PF 2006^[14]^	52	Advanced	Unclear	GP	Unclear	Unclear	Adequate	Unclear	No
Xu G 2006^[15]^	50	Advanced	≥60	TP	Unclear	Unclear	Adequate	Unclear	No
Zhang WB 2006^[16]^	60	Advanced	≥60	GP	Unclear	Unclear	Adequate	Unclear	No
Zhu J 2005^[17]^	51	Advanced	≥60	GP	Unclear	Unclear	Adequate	Unclear	No

### 2.3 疗效与安全性分析结果

#### 2.3.1 疗效*meta*分析比较结果

GP化疗联合热疗对比单纯GP化疗的*meta*分析结果显示，总有效率、生活质量改善率、症状改善率、1年和2年生存率在各研究间的同质性较好（*P* > 0.1, *I^2^* < 50%），均采用固定效应模型，热化组的总有效率、生活质量改善率、症状改善率、1年和2年生存率均好于单化组，其中总有效率、生活质量改善率和症状改善率差异均有统计学意义（*P* < 0.05）；1年和2年生存率的差异无统计学意义（*P* > 0.05），尚需增大样本量进一步验证（图 2，图 3，表 2）。

**2:**
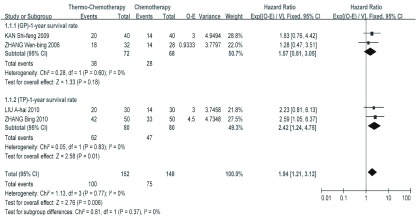
化疗联合热疗组与单独化疗组1年生存率比较的*meta*分析结果 *Meta* analysis of 1-year survival rate for patients with NSCLC treated with Thermo-Chemotherapy and Chemotherapy alone. NSCLC: non-small cell lung cancer.

**3:**
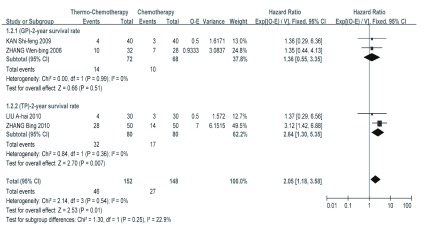
化疗联合热疗组与单独化疗组2年生存率比较的*meta*分析结果 *Meta* analysis of 2-year survival rate for patients with NSCLC treated with Thermo-Chemotherapy and Chemotherapy alone

**2:** 热化组（GP化疗联合热疗）与单独化疗组（GP化疗）疗效与安全性比较的*meta*分析 *Meta* analysis of efficacy and safety for patients with NSCLC treated with GP Thermo-Chemotherapy and GP Chemotherapy alone

Outcome	Included studies	(GP) TCT			(GP) CT			Heterogeneity		Statistical method	Result of *meta*-analysis	
		*n*	N		*n*	N		*I^2^*	*P*		OR (95%CI)	*P*
Total effective rate	7^[3, 4, 10, 11, 14, 16, 17]^	97	227		75	236		0	0.99	OR (M-H, Fixed, 95%CI)	1.69 (1.14-2.52)	0.010
Improving rate of life quality	3^[3, 10, 14]^	82	104		56	108		0	0.59	OR (M-H, Fixed, 95%CI)	3.43 (1.88-6.26)	＜0.0001
Improving rate of symptom	2^[4, 14]^	43	62		29	66		0	0.86	OR (M-H, Fixed, 95%CI)	2.88 (1.39-5.95)	0.004
Myelosuppression	2^[3, 11]^	56	71		59	73		0	0.35	OR (M-H, Fixed, 95%CI)	0.89 (0.39-2.02)	0.78
Gastrointestinal reaction	2^[3, 11]^	50	71		52	73		0	0.76	OR (M-H, Fixed, 95%CI)	0.98 (0.47-2.04)	0.95
Hepatic lesion	2^[3, 11]^	17	71		17	73		0	0.61	OR (M-H, Fixed, 95%CI)	1.02 (0.47-2.24)	0.96
Renal lesion	2^[3, 11]^	11	71		9	73		0	0.43	OR (M-H, Fixed, 95%CI)	1.30 (0.50-3.35)	0.59
GP: gemcitabine and cisplatin; TCT: thermo-chemotherapy; CT: chemotherapy; M-H: *Mantel*-*Haenszel*.												

TP化疗联合热疗对比单纯TP化疗的*meta*分析结果显示，总有效率、生活质量改善率、1年和2年生存率在各研究间的同质性较好（*P* > 0.1, *I^2^* < 50%），均采用固定效应模型，热化组的总有效率、生活质量改善率、1年和2年生存率均好于单化组，且其差异均有统计学意义（*P* < 0.05）（图 2，图 3，表 3）。

**3:** 热化组（TP化疗联合热疗）与单独化疗组（TP化疗）疗效比较的*meta*分析 *Meta* analysis of efficacy and safety for patients with NSCLC treated with TP Thermo-Chemotherapy and TP Chemotherapy alone

Outcome	Included studies	(TP) TCT			(TP) CT			Heterogeneity		Statistical method	Result of *meta*-analysis	
		*n*	N		*n*	N		*I^2^*	*P*		OR (95%CI)	*P*
Total effective rate	8^[5-9, 12, 13, 15]^	137	245		79	244		0	0.93	OR (M-H, Fixed, 95%CI)	2.74 (1.88-4.00)	< 0.000, 01
Improving rate of life quality	5^[5-8, 12]^	102	146		52	142		0	0.95	OR (M-H, Fixed, 95%CI)	4.15 (2.52-6.85)	< 0.000, 01
Myelosuppression	4^[8, 9, 13, 15]^	77	129		83	132		0	0.70	OR (M-H, Fixed, 95%CI)	0.88 (0.53-1.47)	0.63
Gastrointestinal reaction	3^[8, 13, 15]^	65	79		68	82		0	0.84	OR (M-H, Fixed, 95%CI)	0.98 (0.42-2.29)	0.96
Diarrhoea	3^[8, 13, 15]^	21	79		20	82		0	0.82	OR (M-H, Fixed, 95%CI)	1.13 (0.56-2.32)	0.73
Neurotoxicity	3^[8, 13, 15]^	41	79		41	82		0	0.86	OR (M-H, Fixed, 95%CI)	1.14 (0.57-2.31)	0.71
TP: paclitaxel and cisplatin.												

对GP与TP方案亚组间同质性较好（*P* > 0.1, *I^2^* < 50%）的指标进行合并分析，*meta*分析结果显示，热化组的生活质量改善率、1年和2年生存率均好于单化组，且其差异均有统计学意义（*P* < 0.05）（图 2，图 3，表 4）。在*meta*分析中去掉部分质量较差的研究数据做敏感性分析，结果未发生逆转且变化较小，证明了*meta*分析结果的稳健性。

**4:** 化疗联合热疗组与单独化疗组疗效与安全性比较的*meta*分析 *Meta* analysis of efficacy and safety for patients with NSCLC treated with Thermo-Chemotherapy and Chemotherapy alone

Outcome	Included studies	TCT			CT			Heterogeneity		Statistical method	Result of *meta*-analysis	
		*n*	N		*n*	N		*I^2^*	*P*		OR (95%CI)	*P*
Improving rate of life quality	8^[3, 5-8, 10, 12, 14]^	184	250		108	250		0%	0.96	OR (M-H, Fixed, 95%CI)	3.84 (2.61-5.64)	< 0.000, 01
Myelosuppression	6^[3, 8, 9, 11, 13, 15]^	133	200		142	205		0%	0.81	OR (M-H, Fixed, 95%CI)	0.88 (0.57-1.37)	0.58
Gastrointestinal reaction	5^[3, 8, 11, 13, 15]^	115	150		120	155		0%	0.98	OR (M-H, Fixed, 95%CI)	0.98 (0.56-1.71)	0.93
Hepatic lesion	3^[3, 8, 11]^	21	101		20	103		0%	0.83	OR (M-H, Fixed, 95%CI)	1.09 (0.54-2.19)	0.82
Renal lesion	3^[3, 8, 11]^	14	101		14	103		0%	0.48	OR (M-H, Fixed, 95%CI)	1.02 (0.46-2.26)	0.96
Diarrhoea	4^[3, 8, 13, 15]^	30	119		26	122		0%	0.88	OR (M-H, Fixed, 95%CI)	1.26 (0.69-2.31)	0.45

#### 2.3.2 安全性*meta*分析比较结果

GP化疗联合热疗对比单纯GP化疗的*meta*分析结果显示：骨髓抑制、胃肠反应、肝脏损害和肾脏损害在各研究间的同质性较好（*P* > 0.1, *I^2^* < 50%），均采用固定效应模型，热化组与单化组的骨髓抑制、胃肠反应、肝脏损害和肾脏损害发生率差异无统计学意义（*P* > 0.05）（表 2）。3项GP方案的研究^[10, 16, 17]^报道了热疗中因操作不到位而导致皮肤烫伤，但均报道对症处理后短期内痊愈，并不影响治疗进行。

TP化疗联合热疗对比单纯TP化疗的*meta*分析结果显示：骨髓抑制、胃肠反应、腹泻和神经毒性在各研究间的同质性较好（*P* > 0.1, *I^2^* < 50%），均采用固定效应模型，热化组与单化组的骨髓抑制、胃肠反应、腹泻和神经毒性发生率差异均无统计学意义（*P* > 0.05）（表 3）。3项TP方案的研究^[9, 13, 15]^报道了热疗中因操作不到位而导致皮肤烫伤，但均报道对症处理后短期内痊愈，对皮肤烫伤已能较好地预防和治疗。

对GP与TP方案亚组间同质性较好（*P* > 0.1, *I^2^* < 50%）的指标进行合并分析，*meta*分析结果显示热化组与单化组的骨髓抑制、胃肠反应、肝肾损害以及腹泻发生率的差异均无统计学意义（*P* > 0.05）（表 4）。在*meta*分析中去掉部分质量较差的研究数据做敏感性分析，结果未发生逆转且变化较小，证明了*meta*分析结果的稳健性。

#### 2.3.3 发表性偏倚

在本文的*meta*分析中，热化组对比单化组的总有效率在各文献中均有报道，为全面反映纳入研究的情况，最终采用总有效率指标来对纳入文献进行漏斗图分析。漏斗图（图 4）对称性较好，提示存在发表偏倚的可能性较小。

**4:**
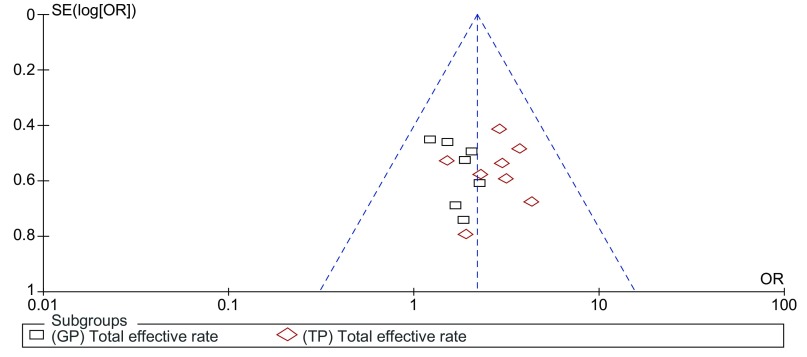
漏斗图分析 Funnel plot analysis

## 3 讨论

### 3.1 本系统评价及纳入研究的局限性

此次研究的局限性：①除1篇SCI的高质量外文文献外，其余中文文献质量一般，限制了本文的证据强度；②各研究间干预措施不尽相同，可能会对结果有一定影响；③生存率及安全性指标的总样本量尚显不足，尚无法对热化联合模式治疗NSCLC的长期疗效和安全性做出非常确定的评价。

系统评价不仅应对现有的临床试验分析评价以指导临床，而且应对未来相关临床研究提出方向性的指导。本文纳入研究的局限性及对以后相关研究的建议：①本文纳入的15项研究，大部分未对随机细节和盲法的实施情况进行报道，希望以后的研究能详细描述随机序列产生方法和随机分配隐藏以及盲法的实施情况；②各RCT的诊治和安全性评价标准不统一，希望以后的研究能控制可能产生偏倚的各种情况，以提高相关研究的准确性并有利于对该方面临床试验结果的评价；③大部分纳入研究缺乏远期疗效和安全性指标的报告，且全部缺乏经济学指标的报道，建议以后的临床试验加强对远期疗效和安全性指标的观测和记录，并收集相关的经济学数据，以实现对热化疗联合疗法的全面评价。

### 3.2 热化联合的疗效及安全性分析

肺癌的发病率和死亡率均在不断上升，而其中80%以上为NSCLC，大部分患者被诊断时已为中晚期，不适合手术治疗。非手术的治疗模式均在探讨中，而化疗联合热疗在理论与临床实践中都显示出良好的应用前景。热化联合治疗并非热疗和化疗的简单相加，而是可以相互协同互补增效，理论上较单纯化疗具有非常明显的优势，能提高化疗疗效并能降低化疗的毒副反应。

以铂类为基础联合第三代化疗药物的两药方案已成为治疗中晚期NSCLC的标准一线方案，吉西他滨（GEM）、紫杉醇（TAX）和长春瑞滨（NVB）是治疗肺癌的一线药物，它们分别与顺铂联合治疗中晚期NSCLC疗效确切^[18]^。国内外多中心随机对照临床研究^[19-21]^表明，GEM、TAX、NVB分别与DDP联合治疗中晚期NSCLC的有效率为30.0%-44.4%，中位生存期为11个月-17个月，1年生存率为35.0%-61.0%，差异均无统计学意义。研究小组已完成了对NP方案热化疗的*meta*分析^[22]^，本文则探讨了GP与TP方案热化疗的临床价值。

吉西他滨（GEM）是治疗NSCLC较为有效的药物之一，主要作用于DNA合成期（S期），也可以阻止细胞由G_1_期向S期转化，是细胞周期特异性药物，可以完全抑制DNA链继续延长从而引起细胞凋亡^[23]^。紫杉醇是一种新型植物性抗肿瘤药物，主要作用于癌细胞的微管蛋白系统。该药通过促进微管蛋白聚合并抑制其解聚，使细胞分裂停止于G_2_/M期，从而阻止了肿瘤细胞的增殖。而顺铂（DDP）是细胞周期非特异性药物，可作用于细胞周期的任一时相。目前，以铂类为基础联合吉西他滨或紫杉醇的两药化疗方案已成为治疗中晚期NSCLC的一线标准方案，对中晚期患者有利于控制病灶的扩散，有效改善临床症状，提高生活质量，且毒副反应较轻，患者均能耐受。

热化疗协同抗癌机制：①热化疗有利于化疗药物进入癌细胞；②热增加药物与DNA交联，增强对癌细胞的杀伤；③热能抑制化疗后癌细胞DNA的修复和合成以及耐药基因表达，增加癌细胞对化疗药的敏感性，逆转某些化疗药物耐药^[24]^；④热化疗促使癌细胞凋亡发生；⑤对乏氧细胞和富氧细胞以及对肿瘤组织中心部位和周边部位^[25]^，热疗和化疗的抗癌有互补增效的作用。此外，局部热疗联合全身化疗兼顾局部治疗与全身治疗，在增强局部治疗强度同时消灭远处微转移病灶，发挥协同抗肿瘤作用，提高疗效，降低单用剂量从而减轻毒副作用，提高患者耐受性。

本系统评价结果显示：GP或TP方案化疗联合热疗治疗不宜手术的中晚期NSCLC患者，能提高近期疗效和远期生存率，改善症状和生活质量，毒副反应主要为化疗所致，未见热疗引起的明显不良反应，且热疗有降低化疗毒副反应的趋势。NP方案热化疗的*meta*分析^[22]^及本文GP和TP方案热化疗的*meta*分析都表明，化疗联合热疗是一种安全、合理、有效的治疗方法，可以作为一线方案推荐临床使用，使广大患者从中获益，但远期疗效有待于大样本RCT进一步证实。

关于化疗联合热疗在临床实践中的具体实施操作还有很多尚待解决的问题。热化联合治疗的时间顺序尚无定论，但有实验表明，当加热与给药同时进行时，对细胞的毒性作用最大，所以一般认为，同时治疗的效果较强。热疗应当在肿瘤组织中药物浓度达到最高时进行，对快速静脉给药而言，应在给药完毕后立即进行热疗；对缓慢静脉给药而言，肿瘤中药物浓度高峰在注射时的中间或后1/3段时出现，此时热疗最好^[26]^。此外，热化疗各自的方式和组配剂量以及如何在提高疗效的同时进一步降低毒副反应，都是临床研究中需要继续探讨的问题，希望在以后的临床研究中能有较好的体现。总之，为获得热化疗联合治疗NSCLC更为精确而全面的临床疗效和安全性以及经济学评价以便更好地指导临床决策，尚需进行更多设计、执行和报告均良好的高质量随机对照试验。

热疗作为并列于手术、放疗和药物的肿瘤疗法，已在肿瘤的治疗研究中广泛展开，取得了令人鼓舞的成绩，国内外大量相关的高质量循证研究都证实了肿瘤热疗的临床价值^[27-31]^。然而目前热疗的临床技术还并不成熟，还不能实现完美到位的热疗，以致影响了热疗的效果，甚至使人们对肿瘤热疗本身产生了怀疑。目标肿瘤部位的加热如不经过严格的质量保证，并不总能如预期那样达到令人满意的疗效。如何实现热疗的靶向治疗和无损测温、如何降低或消除热耐受现象的影响以及热剂量的选择控制，都是决定热疗临床效果的关键问题，也是目前热疗发展的瓶颈，希望在以后的基础和临床研究中能实现突破性进展。随着现代科技的进步，相信热疗会在今后肿瘤的综合治疗中发挥更加重要的作用。

## References

[1] Jemal A, Bray F, Center MM (2011). Global cancer statistics. CA Cancer J Clin.

[2] Wust P, Hildebrandt B, Sreenivasa G (2002). Hyperthermia in combined treatment of cancer. Lancet Oncol.

[3] Shen H, Li XD, Wu CP (2011). The regimen of gemcitabine and cisplatin combined with radio frequency hyperthermia for advanced non-small cell lung cancer: a phase Ⅱ study. Int J Hyperthermia.

[4] Zhang GZ, Nan H, Chao LN (2011). Study on the effect of GP regimen combined with thermotherapy for advanced non-small cell lung cancer. Yi Xue Xin Xi.

[5] Cheng P (2010). Effect of TP chemotherapy combined with thermotherapy for advanced non-small cell lung cancer. Zhongguo Shi Yong Yi Kan.

[6] Liu AH, Li BQ, Li FQ (2010). Effect of hyperthermotherapy combined with TP chemotherapy in treating advanced non-small cell lung cancer. Zhongguo Ai Zheng Fang Zhi Za Zhi.

[7] Wang M (2010). Clinical analysis of hyperthermia combined with TP chemotherapy for advanced non-small cell lung cancer. Hainan Yi Xue.

[8] Yang MX, Zhao J, Wang YW (2010). BSD2000 deep hyperthermia combined with chemotherapy of PT regimen in patients with non-small cell lung cancer. Zhongguo Fei Ai Za Zhi.

[9] Zhang B, Tang Y, Xie J (2010). Effect of thermotherapy combined with chemotherapy in treating advanced non-small cell lung cancer. Shandong Yi Yao.

[10] Kan SF, Tian T (2009). Clinical observation of hyperthermia combined with gemcitabine in the treatment of old patients with advanced non-small cell lung cancer. Zhongguo Zhong Liu Lin Chuang Yu Kang Fu.

[11] Cao YL, Hu XH, Lu YK (2008). Clinical study of chemotherapy combined with whole body microwave hyperthermia in treatment of advanced non-small cell lung cancer. Zhonghua Zhong Liu Fang Zhi Za Zhi.

[12] Zhang L, Wu J, Li P (2008). Observational study on the effect of treating advanced NSCLC using TP chemotherapy and thermotherapy. Zhong Liu Yu Fang Yu Zhi Liao.

[b13] 13Zhou M. Effects of 41. 8 ℃ whole-body hyperthermia on cellular immune function of patients with non-small cell lung cancer. 2007.http://www.europepmc.org/abstract/MED/10873112周明. 41. 8 ℃全身热疗对非小细胞肺癌患者细胞免疫功能的影响. 2007.

[14] Chen PF, Zheng LF (2006). Nursing research of hyperthermia combined with chemotherapy for advanced non-small cell lung cancer. Zhongguo Zhong Yi Yao Xian Dai Yuan Cheng Jiao Yu.

[15] Xu G, Wang YD, Zhou M (2006). Whole body hyperthermia combined with chemotherapy for the treatment of advanced non-small cell lung cancer. Zhong Liu Yan Jiu Yu Lin Chuang.

[16] Zhang WB, Xie TF, Xie HS (2006). Clinical observation of hyperthermia combined with chemotherapy in the treatment of advanced non-small cell lung cancer. Zhong Liu.

[17] Zhu J, Hou M, Cao D (2005). Hyperthermia combined with chemotherapy in the treatment of advanced non-small cell lung cancer: an initial study. Zhongguo Fei Ai Za Zhi.

[18] Liu L, Wang XW, Li L (2006). A Randomized comparative trial of three combined regimens containing cisplatin for treatment of advanced non-small cell lung cancer. Ai Zheng.

[19] Bretti S, Manzin E, Loddo C (2002). Gemcitabine plus cisplatin in the treatment of patients with advanced non-small cell lung cancer: a phase Ⅱ study. Anticancer Res.

[20] Chen CH, Chang WC, Lin MC (2002). Phase Ⅱ study of paclitaxel (Genaxol) and cisplatin combination in treating Chinese patients with advanced non-small cell lung cancer (NSCLC). Lung Cancer.

[21] Le Chevalier T, Brisgand D, Pujol JL (1996). Results of a randomized study comparing combination of navelbine-cisplatin to combination of vindesine-cisplatin and to navelbine alone in 612 patients with inoperable non-small cell lung cancer. Bull Cancer.

[22] Li Z, Mi DH, Yang KH (2011). Thermotherapy plus NP for patients with non-small cell lung cancer: a *meta*-analysis. Gansu Yi Yao.

[23] Barton-Burke M (1999). Gemcitabine: a pharmacologic and clinical overview. Cancer Nurs.

[24] Westermann AM, Grosen EA, Katschinski DM (2001). A pilot study of whole body hyperthermia and carboplatin in platinum-resistant ovarian cancer. Eur J Cancer.

[25] Takahashi I, Emi Y, Hasuda S (2002). Clinical application of hyperthermia combined with anticancer drugs for the treatment of solid tumors. Surgery.

[26] Parks LC, Minaberry D, Smith DP (1979). Treatment of far-advanced bronchogenic carcinoma by extracorporeally induced systemic hyperthermia. J Thorac Cardiovasc Surg.

[b27] 27Lutgens L, van der Zee J, Pijls-Johannesma M, *et al*. Combined use of hyperthermia and radiation therapy for treating locally advanced cervical carcinoma. Cochrane Database Syst Rev, 2010 Jan 20, (1): CD006377.http://europepmc.org/abstract/MED/20091593

[28] Mi DH, Li Z, Yang KH (2011). HRCT for non-small cell lung cancer: a *meta*-analysis. Zhongguo Xun Zheng Yi Xue Za Zhi.

[29] Yan TD, Black D, Sugarbaker PH (2007). A systematic review and *meta*-analysis of the randomized controlled trials on adjuvant intraperitoneal chemotherapy for resectable gastric cancer. Ann Surg Oncol.

[30] Gill RS, Al-Adra DP, Nagendran J (2011). Treatment of gastric cancer with peritoneal carcinomatosis by cytoreductive surgery and HIPEC: a systematic review of survival, mortality, and morbidity. J Surg Oncol.

[31] Chua TC, Robertson G, Liauw W (2009). Intraoperative hyperthermic intraperitoneal chemotherapy after cytoreductive surgery in ovarian cancer peritoneal carcinomatosis: systematic review of current results. J Cancer Res Clin Oncol.

